# Successful administration of chimeric antigen receptor (CAR) T-cell therapy in patients requiring hemodialysis

**DOI:** 10.1186/s40164-022-00266-1

**Published:** 2022-02-28

**Authors:** Bradley D. Hunter, Daanish Hoda, Andy Nguyen, Launce Gouw, Bryan Huber, Ryan R. Jensen, Justine Preedit, Andrew Evens, Esther Huang, Jiyeon Park, Dennis L. Cooper

**Affiliations:** 1 Blood and Marrow Transplant, Intermountain Healthcare, LDS Hospital, 8th Avenue and C Street, E8 BMT, Salt Lake City, UT United States; 2 Blood and Marrow Transplantation, Rutgers Cancer Institute of New Jersey, New Brunswick, NJ United States

## Abstract

Chimeric antigen receptor (CAR) T-cell therapy has revolutionized the treatment of relapsed/refractory B-cell malignancies. However, there is no data on the safety and efficacy of CAR T-cell therapy in patients with end stage renal disease (ESRD) requiring dialysis. In this report, we present two patients with DLBCL and ESRD who were successfully treated with different CAR T-cell products. Patient #1 is a 66 year-old woman with a history of HIV who was treated to complete response with axicabtagene ciloleucel with treatment complicated by grade 1 cytokine release syndrome (CRS) and grade 2 immune effector cell-associated neurolotoxicity syndrome (ICANS). Patient #2 is 52 year old woman whose ESRD was caused by ifosphamide toxicity and was treated to complete response with lisocabtagene maraleucel and did not experience either CRS or ICANS. Both patients received lymphodepletion chemotherapy with fludarabine and cyclophosphamide, which was dose-adjusted for ESRD with scheduled dialysis 12 h after each dose of lymphodepletion chemotherapy. Patients with DLBCL and ESRD can be safely administered both lymphodepletion chemotherapy and CAR T-cell therapy. Additionally, the fact that both patients achieved complete response to therapy suggests that CAR T-cell therapy should be strongly considered in patients with ESRD. Long-term follow up is needed to determine if therapy in this setting is of curative intent.

**To the Editor**,

Chimeric antigen receptor (CAR) T-cell therapy is a revolutionary treatment modality for relapsed/refractory B-cell malignancies. Historically, the median survival for patients with relapsed/refractory DLBCL after 2 lines of therapy was 6 months, and only 7% of patients were able to attain complete remission (CR) to the next line of therapy [1].

Both clinical trial and off-trial (so-called “real world”) experience with CAR T-cell therapy for B-cell malignancies have yielded impressive results [2–4] with reports showing 44% overall survival at 4 years post-CAR T infusion [5]. However, data is still lacking for patients receiving dialysis for ESRD. This is due to patients with ESRD being excluded from CAR T trials to date. Apart from the issue of tolerance of CAR T cells in patients receiving renal replacement therapy, there is the potential for enhanced toxicity after lymphodepleting treatment related to poor clearance of fludarabine [6–9]. Previous reports show that fludarabine as a conditioning regimen prior to stem cell transplant in patients with ESRD requiring dialysis is possible [10–12].

We report the successful treatment of 2 dialysis-dependent patients with CAR T-cell therapies (axicabtagene ciloleucel and lisocabtagene maraleucel) for relapsed/refractory DLBCL.

Patient #1 was a 66 year old woman with HIV who developed idiopathic ESRD in 2015. In April 2018, she presented with diffuse cervical lymphadenopathy. Biopsy showed DLBCL, ABC subtype. PET CT showed diffuse FDG avid disease without extranodal involvement. She was initially treated with R-EPOCH but switched to R-CHOP because of toxicity and completed six cycles of therapy with intrathecal prophylaxis. A PET CT showed CR but 18 months later had biopsy-confirmed recurrence in the neck, abdomen and spleen. She was treated with prednisone and vincristine with excellent response, followed by gemcitabine and oxaliplatin. PET CT showed metabolic CR. She was felt to be a better candidate for CAR T cell therapy rather than autologous stem cell transplant [13]. Of note, her HIV viral load was undetectable prior to apheresis, and her CD4 count was 629/mcl.

The patient was admitted for lymphodepleting chemotherapy (LDC), and received cyclophosphamide 300 mg/m^2^ on days -5, -4, and -3, and fludarabine 20 mg/m^2^ on days -5, -4, and -3 (see Fig. 1); fludarabine was dose reduced from 30 mg/m^2^ due to concomitant dialysis. Hemodialysis was timed to be performed 12 h following each dose of fludarabine to minimize toxicity [10–12]. After completion of LDC, she resumed her previous schedule of intermittent dialysis. She was treated with axicabtagene ciloleucel with a course complicated by grade 1 CRS (day + 6) and grade 2 ICANS which required ICU-level care. She received solumedrol 1 gm daily × 2 days with excellent response followed by steroid taper. She was discharged on day + 15. Four months after CAR T cell therapy, she had new neurologic symptoms and was found to have brain parenchyma recurrence without systemic disease. She died shortly thereafter.

**Fig. 1:**
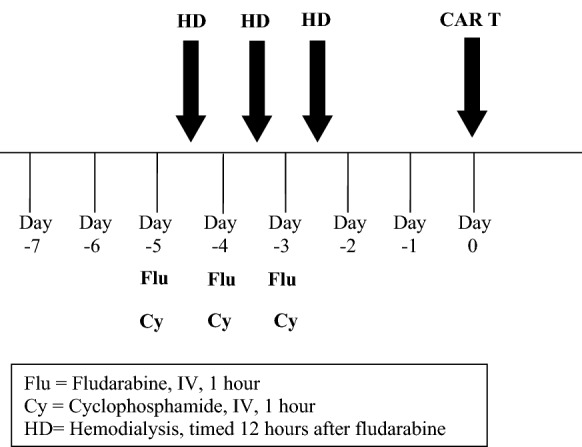
Lymphodepletion regimen for patients with ESRD requiring hemodialysis

Patient # 2 is a 52-year-old woman who was diagnosed with T-cell histiocyte-rich DLBCL and treated to complete response (CR) with R-CHOP chemotherapy 11 years prior to subsequent relapse. She was then treated with R-ICE salvage, complicated by ifosfamide toxicity (encephalopathy and renal failure), requiring hemodialysis. Her encephalopathy resolved and she achieved partial response, but remained dialysis-dependent. She was then treated with nivolumab (PD-L1 expression 81–90%) for 8 weeks and achieved CR. Nivolumab treatment was complicated by rash to 91% of her body surface area, diarrhea, pneumonitis, and cardiomyopathy. These symptoms resolved with steroids (including normalization of her LVEF), but treatment was not resumed due to the severity of toxicity. Her disease again relapsed, and she was treated with tafasitamab + lenalidomide. Cycle 1 was interrupted due to intractable diarrhea from either current therapy or recrudescence of immune-mediated colitis. After 2 cycles, PET CT showed progression of disease. Lenalidomide was continued, but tafasitamab was held for 5 weeks prior to lisocabtagene maraleucel therapy. The patient’s PET CT just prior to liso-cel infusion was notable for a partial response.

Lymphodepleting therapy and dialysis was managed as in patient #1. Lisocabtagene maraleucel was administered in the outpatient setting. The patient did not experience CRS or immune effector cell-associated neurotoxicity syndrome (ICANS). PET CT on day 28 as well as repeat PET CT 6 months post infusion were notable for CR. She is now > 9 months from infusion and in ongoing CR.

The treatment courses of these patients demonstrate that both lymphodepleting chemotherapy and CAR T-cell therapy (axi-cel and liso-cel) can be safely administered to patients with ESRD. One patient remains in metabolic CR at 9 months; however, further follow-up and more patients will be required to better determine long term efficacy.

## Data Availability

The datasets used and/or analysed during the current study are available from the corresponding author on reasonable request.
